# Updated Views on Vestibular Physical Therapy for Patients with Vestibular Disorders

**DOI:** 10.3390/healthcare13050492

**Published:** 2025-02-24

**Authors:** Marco Tramontano, Souad Haijoub, Michel Lacour, Leonardo Manzari

**Affiliations:** 1 Department of Biomedical and Neuromotor Sciences (DIBINEM), Alma Mater University of Bologna, 40126 Bologna, Italy; 2 Unit of Occupational Medicine, IRCCS Azienda Ospedaliero-Universitaria di Bologna, 40126 Bologna, Italy; 3 Independent Researcher, 75015 Paris, France; souad.haijoub@gmail.com; 4 Neurosciences Department, Aix-Marseille University, CNRS, 13005 Marseille, France; michel.lacour0802@gmail.com; 5 MSA ENT Academy Center, 03043 Cassino, Italy; lmanzari1962@gmail.com

**Keywords:** vestibular physical therapy, vestibular compensation, restoration, substitution, habituation

## Abstract

**Background/Objectives:** Vestibular Physical Therapy (VPT) plays a crucial role in the rehabilitation of patients with vestibular disorders by promoting vestibular compensation through adaptation, habituation, and substitution mechanisms. While traditional VPT approaches have demonstrated effectiveness in restoring balance and gaze stability, some patients with chronic vestibular dysfunction continue to experience persistent deficits. **Methods:** review of recent advancements in neuroplasticity research suggest the need for updated rehabilitation strategies that integrate sensorimotor substitution, saccadic training, optokinetic stimulation, and cognitive–motor dual-task training to optimize vestibular compensation. **Results:** This perspective article explores innovative VPT approaches aimed at improving dynamic gaze and postural stability in a more challenging way. We emphasize the importance of personalized rehabilitation strategies that leverage multisensory integration to enhance neuroplastic recovery. **Conclusions:** By refining VPT interventions, we can maximize functional outcomes and improve the quality of life for individuals with vestibular disorders.

## 1. Introduction

Vestibular disorders encompass conditions that affect the inner ear and brain, resulting in ocular motor symptoms (nystagmus, oscillopsia, blurred vision), postural and locomotor symptoms (instability, dizziness, gait deviation), and perceptive (vertigo, spatial orientation) and cognitive (attention, spatial memory, navigation) symptoms [1,2,3,4,5]. These symptoms significantly reduce quality of life and burden healthcare systems worldwide [6]. Common vestibular disorders include benign paroxysmal positional vertigo (BPPV), vestibular migraine, Meniere’s disease, unilateral vestibulopathy, and bilateral vestibulopathy [2]. Millions of people suffer from these conditions, and their prevalence increases with age, disproportionately affecting the elderly population [7]. Rehabilitation plays a critical role in the recovery process for individuals with vestibular disorders [8]. While acute conditions may resolve spontaneously or improve with early mobilization [9,10], chronic vestibular dysfunction often requires targeted interventions to restore balance and gaze stability, reduce symptoms, and prevent falls [11,12,13]. Persistent symptoms may result from incomplete peripheral recovery, poor central compensation, or maladaptive strategies [14]. Moreover, psychiatric comorbidities, such as anxiety and depression, exacerbate the subjective experience of these disorders [15,16].

Vestibular Physical Therapy (VPT) has been the cornerstone of the management of vestibular disorders for decades [17,18]. Initially developed in the mid-20th century by Cawthorne and Cooksey, traditional VPT is believed to interact with the vestibular lesion-induced neuronal plasticity, and it is designed to promote vestibular recovery or compensation through exercises targeting gaze stabilization and balance training [19]. These exercises aim to improve the impaired vestibular functions particularly by stimulating the ocular and postural control mechanisms, to improve gaze stability and reduce instability and dizziness, and to restore a good quality of life [17,18,19].

Traditionally, VPT focuses on three primary principles [17]:

Adaptation involves prolonged adjustments in the vestibular system’s neuronal firing rate in response to head movements, aiming to minimize retinal slip [20]. Adaptation exercises require active head movements while keeping the gaze fixed on a target, which can be either stationary or in motion.

Habituation is the process of diminishing a behavioral response after repeated exposure to a provoking stimulus, aimed at alleviating vestibular-related symptoms [21].

Substitution consists of exercises aiming to promote alternative strategies (e.g., preprogramming of eye movements) that substitute for missing vestibular functions [22,23].

However, despite the effectiveness of traditional approaches, many patients—particularly those with chronic vestibular disorders—continue to experience incomplete recovery. These individuals often exhibit persistent balance and gaze control deficits, which prevent them from returning to full function in dynamic, real-world environments. The recovery of static vestibular functions (i.e., those observed at rest) occurs relatively quickly, but the recovery of dynamic vestibular functions, such as the VOR during head movements or balance control during dynamic tasks, is generally slower and often incomplete.

## 2. Rationale for Updated Views on Vestibular Physical Therapy

**Figure 1:**
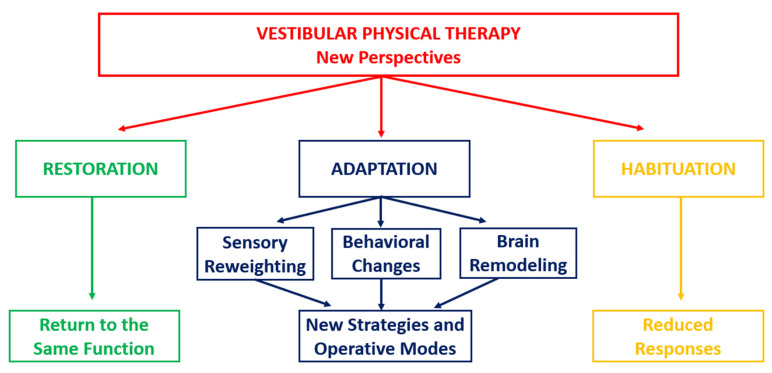
Flow diagram for new strategies in Vestibular Physical Therapy.

Several studies focused on vestibular recovery in animal models have provided a robust theoretical framework for understanding the compensation mechanisms following vestibular dysfunction [24,25]. According to these studies, improvements in vestibular function rely on the three key mechanisms previously defined: **restoration**, **adaptation** via sensorimotor substitution, and **habituation**. These mechanisms are part of the brain’s neuroplastic response to vestibular loss. They drive full synaptic remodeling of the pathways (restoration); functional reorganization of neuronal networks through the use of sensorimotor strategies drawn from the behavioral repertoire, or by creating new substituting modes (adaptation); and an improvement in the ability to ignore or reduce the response to disturbing stimuli (habituation). All these mechanisms reflect the brain’s orchestration of neuronal network remodeling, which represents vicariant idiosyncratic melodies played by each vestibular patient, as illustrated in Figure 1.

This is important for the often-blurred link with other forms of vestibular dysfunction that present a more central origin. Indeed, the clinical similarity between vestibular disorders and neurological diseases (i.e., vestibular migraine and episodic type 2 ataxia) suggests a shared pathophysiological mechanism [26]. Furthermore, certain vestibular disorders exhibit age-dependent modifications. The cerebellum, characterized by a high density of calcium channel receptors, may further contribute to the blurred distinctions between peripheral and central vestibular dysfunctions. This highlights the importance of age-specific considerations in diagnosis and treatment planning [27].

**Restoration** involves regaining the original vestibular functions. It refers to the spontaneous or VPT-induced recovery of lost vestibular functions using the same neural pathways that were in place before the injury.

**Adaptation** involves enhancing the vestibular system’s response to stimuli, either by employing alternative sensory inputs (sensory substitution) or by elaborating new strategies (behavioral substitution) to compensate for the lost vestibular functions. It occurs when other sensory modalities, such as vision or proprioception, or newly operating modes, such as the ocular saccades, take over the role of the vestibular system. This is particularly important for dynamic functions, for which multisensory integration compensates for the ongoing deficits in vestibular input and behavioral substitution strategies that are crucial to improving daily activities.

**Habituation** involves reducing the brain’s sensitivity to repetitive vestibular stimuli through desensitization. It is the gradual decline of inappropriate responses to repetitive sensory–motor stimuli. Over time, exposure to certain movements or stimuli helps desensitize the vestibular system, thereby reducing symptoms such as dizziness and blurred vision.

Neural plasticity is the key mechanism driving these compensatory strategies, and brain-plasticity-based therapeutics are the way to normalize defective vestibular functions [28,29,30]. Indeed, neuroplastic changes within the vestibular nuclei, vestibulo-cerebellum, and parieto-insular vestibular cortex play a pivotal role in recovery. These changes include synaptic remodeling and the functional reorganization of neural networks, facilitated by vestibular rehabilitation exercises that target sensory and motor integration [31].

The recovery process involves both structural and functional reorganization within the central nervous system (CNS). For example, after unilateral vestibular loss, plastic changes occur in the vestibular nuclei and associated pathways, including the vestibulo-cerebellum and the parieto-insular vestibular cortex [31]. These changes are impacted by VPT exercises that facilitate restoration or functional compensation of the vestibulo-ocular reflex (VOR) through catch-up and covert saccades [32,33].

Given the limitations of traditional VPT in addressing dynamic vestibular deficits, newer therapeutic approaches are needed to further enhance the neuroplastic processes that support vestibular compensation. This manuscript explores innovative rehabilitation strategies focusing on more comprehensive and personalized rehabilitation, particularly for patients who have not responded adequately to traditional methods. We suggest that the type of vestibular lesion determines the nature of the induced recovery mechanisms [34]; for this reason, only after an instrumental evaluation of all the vestibular receptors will it be possible to plan the proper training.

## 3. New Perspectives

### 3.1. Gaze Stability Training

Sensory substitution is a powerful mechanism within the vestibular compensation process, allowing the brain to rely on other sensory inputs, such as vision and proprioception, when vestibular signals are unavailable or insufficient. This concept has been revisited in recent years in response to advancements in instrumental assessments, enhancing the efficacy of personalized VPT.

**Figure 2:**
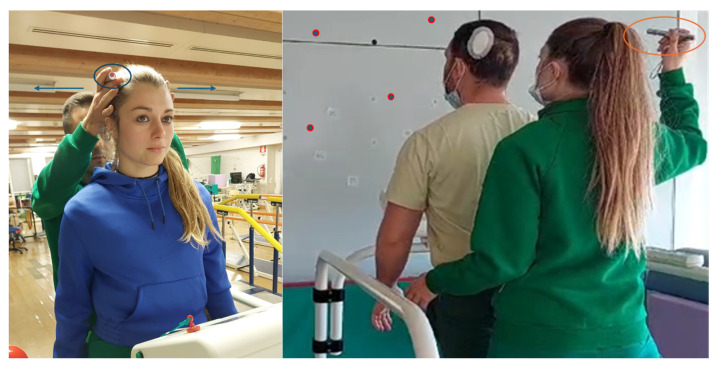
Dynamic–unpredictable saccadic training. On the left side of the figure, substitution training using SHIMP maneuvers is shown to enhance passive, unpredictable, and rapid saccades. The patient is asked to follow a red dot on the wall, generated by a laser attached to the top of the head, while the clinician delivers unpredictable head impulses. On the right side, unpredictable saccadic movements are stimulated by the physiotherapist, who manually directs a handheld laser that the patient must follow during gait with rapid head movements. The patient combines horizontal and vertical movements of both the eyes and the head to track the red dot during dynamic tasks.

Saccades are a primary sensory substitution strategy allowing the eyes to quickly reorient to a target when the VOR is impaired. In patients with vestibular hypofunction, saccades help to stabilize gaze during head movements. Saccade training leverages this natural compensatory mechanism to enhance gaze stability during dynamic tasks. The difference with the previous principles is that saccadic movements should be performed under passive and unpredictable head movements during dynamic motor tasks. Another possible strategy is to perform the SHIMPS maneuvers with a laser on the head to train saccadic movements in different dynamic conditions (i.e., in a standing position, walking, jumping) as reported in Figure 2.

### 3.2. Improvement of Dynamic Visual Acuity (DVA)

**Figure 3:**
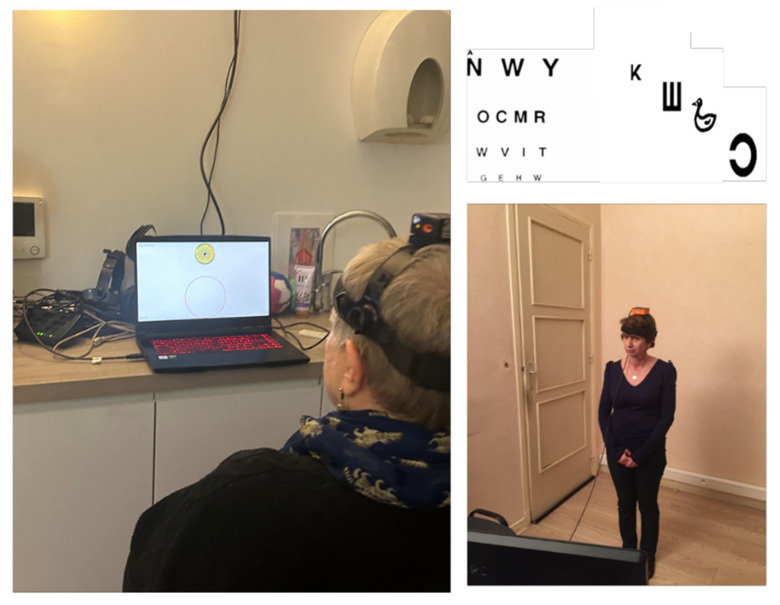
A dynamic visual acuity protocol for rehabilitation. The test can be performed actively by patients sitting in front of a laptop screen or standing in front of a high-resolution screen on which optotypes adapted for adults or young children are randomly presented for a duration of 50 ms. When recognized, the optotype size is then decreased in steps corresponding to changes of 1/10 on the Snellen visual acuity chart.

Decrements in DVA lead to serious problems impacting the patient’s quality of life, including avoidance of driving, difficulties in reading and watching TV, reduced activity levels, and social isolation. Training the patient to perform rapid head rotations in the direction of the deficient VOR contributes to gaze stabilization and improved DVA. Recent findings showed that early DVA protocols are necessary for a rapid recovery of DVA [35]. Moreover, active exercises where patients have to read while moving their head horizontally or vertically constitute good training to improve DVA (see Figure 3).

Gaze stability training can lead to a full recovery of the dynamic canal function (VOR restoration) when two conditions are fulfilled: early active training and the presence of some remaining function in the canal (VOR gain > 0.20) [32]. 

### 3.3. Substitution with Optokinetic Nystagmus (OKN)

**Figure 4:**
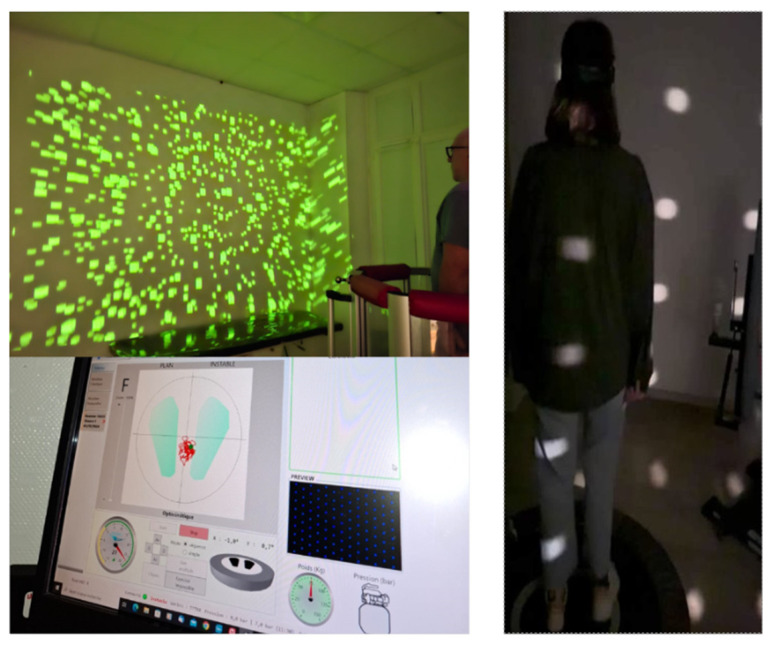
An experimental setup for optokinetic stimulation. Vestibular patients are asked to keep their balance on a posturography platform while submitting to an optokinetic stimulation made of white (right part) or colored (upper left part) dots moving in different planes at various velocities (20–100°/s), depending on the vestibular pathology. The center of foot pressure (lower left part) is shown as the red pathway of the stabilogram (between the two feet) together with the symbols used by the software to measure the recording time and the conditions used during the recording session, i.e., in both static (stable support) and dynamic (unstable support) conditions.

OKN plays a crucial role in dynamic gaze stability. OKN is a reflexive eye movement that occurs in response to large-scale visual motion across the retina, and it is closely linked with vestibular function because it helps stabilize the visual field during head or body movements. The OKN system complements the VOR by stabilizing images on the retina during slow and prolonged head movements. When the vestibular system is damaged, the brain relies more heavily on OKN to maintain stable vision. This interaction between the visual and vestibular systems is part of a broader sensory reweighting strategy. Optokinetic stimuli are often used in rehabilitation settings to train the visual system to compensate for vestibular deficits. Patients are exposed to rotating visual patterns, such as stripes or dots moving across a screen, to elicit OKN and stimulate visual–vestibular integration. Over time, this exposure helps patients adapt to their vestibular loss by enhancing their ability to use visual cues to maintain balance and stabilize their gaze. The incorporation of optokinetic training into vestibular rehabilitation programs can significantly enhance outcomes, especially for patients with incomplete vestibular compensation. This type of training is typically performed using optokinetic drums that simulate complex, moving visual fields. The goal is to stimulate the OKN reflex while the patient performs dynamic tasks or engages in head movements, promoting better integration between visual and vestibular inputs. One goal of optokinetic stimulation is to reduce the vestibular asymmetry observed acutely after symptom onset by favoring visual substitution. In this case, the stimulation must be applied at low velocity (~20°/s). A second goal is to reduce the visual dependency found in chronic vestibular patients or in subjects complaining of highway syndrome. This desensitization (or habituation) protocol can use optokinetic stimulation of up to 100°/s (see Figure 4).

### 3.4. Postural Stability Training

Dynamic postural stability is crucial for patients with vestibulo-spinal impairments, where maintaining balance during motion becomes particularly challenging. Traditional VPT often focuses on static balance training, but recent research highlights the importance of dynamic postural stability exercises, which aim to improve balance during movement. Dynamic postural stability training consists of exercises of habituation and sensory reweighting strategies. Sensory reweighting involves the dynamic adjustment of the central nervous system’s reliance on visual, proprioceptive, and vestibular inputs to maintain postural stability. This process is critical for patients with vestibular deficits as it enables compensation for unreliable vestibular input by enhancing reliance on other sensory modalities. A recent study highlighted the role of multisensory integration in recalibrating balance mechanisms, particularly through targeted training protocols [36].

Dynamic postural stability training involves sensory reweighting, a process by which the brain adjusts its reliance on visual, proprioceptive, and vestibular inputs to maintain balance [37,38]. Sensory reweighting is especially important in patients with vestibular deficits, as it allows them to compensate for unreliable vestibular input by increasing their reliance on other sensory modalities [21].

**Figure 5:**
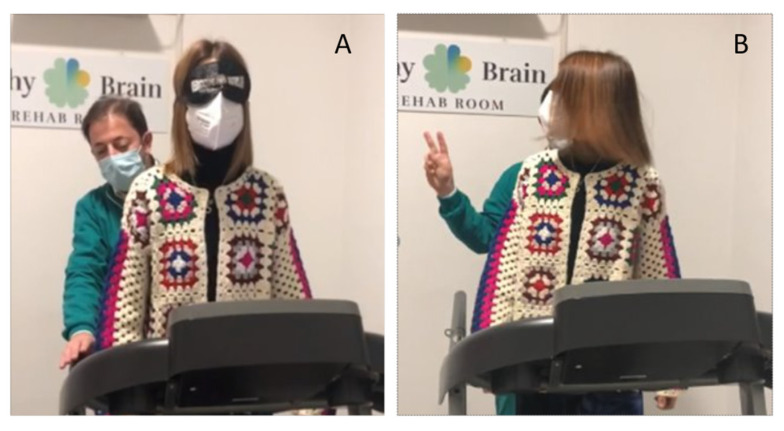
Dynamic blindfolded balance training and a cognitive–motor dual task. In the left part (**A**), blindfolded balance training is shown to enhance dynamic postural stability. The patient is blindfolded and instructed to walk on a treadmill without hand support. In the right part (**B**), cognitive–motor therapy is performed, where the participant engages in a dual-task paradigm: responding to unpredictable auditory stimuli by rotating their head toward the sound. In a variant of this task, they may also have to identify a visual target. These tasks are performed while walking on an unstable surface or treadmill.

Blindfolded balance training on a treadmill is a perturbation-based stimulation during linear acceleration without visual feedback or hand support. This training helps patients to maintain balance in a controlled environment that combines habituation with sensory reweighting training (see Figure 5). This approach enhances not only postural control but also gait performance, improving gait parameters [39].

The rotatory chair protocol is currently used in some countries, but it had not been evaluated prior to recent investigations in patients with acute peripheral unilateral vestibular loss [40]. Repeated whole-body rotations to the lesioned side reduced the vestibular asymmetry and decreased the directional preponderance, suggesting a faster recovery of the electrophysiological homeostasis in the vestibular nuclei. This passive protocol must be performed early after symptom onset and during a short time period to quickly rebalance the vestibulo-spinal outputs on the posture control mechanisms. Patients can more easily perform active exercises just after the protocol in more challenging conditions (i.e., unstable support and/or with eyes closed).

Cognitive–motor dual-task training reflects the complexity of real-world environments. In everyday life, individuals often perform multiple tasks simultaneously, such as walking while talking. Cognitive–motor dual-task training improves a patient’s ability to manage these real-world, dual-task scenarios.

The training consists of a dual-task paradigm in which each patient is asked to walk blindfolded without stopping. It is explained to them that, during the task, they might hear a sound; in that case, they should turn their head towards the side of the stimulus [41]. This dual task can be performed by marching on an unstable surface or by walking on a treadmill at different velocities. Furthermore, this training can be performed to stabilize the gaze in an open eye condition by asking the patient to recognize a visual target when they turn their head.

### 3.5. Perceptive Tasks (The Subjective Visual Vertical)

The perception of verticality is a multisensory integration process elaborated in high-level brain areas (parieto-insular cortex). Many patients with unilateral vestibular loss show a strong bias in their perception of verticality, as assessed by determining the subjective visual vertical (SVV), a test of the otolith system. Without rehabilitation, the recovery of earth-vertical orientation takes several months. However, when patients are trained to stand upright on a tilted support in the roll plane to their disease side, a fast SVV normalization is observed [42]. The rationale of this VPT protocol is to modify the patient’s body posture on the opposite side with respect to the SVV offset in order to increase the weight of the body sensors and to re-build a correct perception of verticality, restoring orientation constancy. A shift to a reference frame based on body-in-space orientation, that is, on an egocentric reference frame, is most often used to recalibrate the perception of verticality and to normalize the SVV. This protocol is also valid for rehabilitating elderly people with instability.

**Figure 6:**
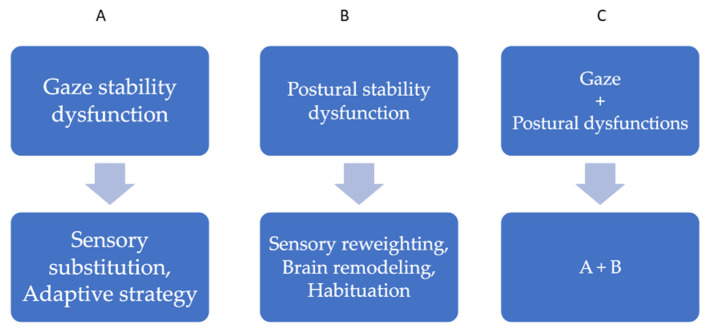
Decisional diagram for rehabilitative processes.

The decisional flow diagram presented in Figure 6 outlines the rehabilitative processes for vestibular dysfunction, categorizing gaze and postural stability impairments and highlighting targeted therapeutic strategies to enhance compensation and recovery.

## 4. Techniques to Improve Activities of Daily Living

The ultimate goal of VPT is to restore a patient’s ability to perform daily activities with minimal limitations. Incorporating exercises into routine tasks helps patients maintain the progress made during formal therapy sessions.

Incorporating vestibular stimulation in naturalistic environments can enhance the efficacy of rehabilitation by leveraging real-world sensory experiences, thereby promoting neural plasticity and improving patient outcomes [43].

- Practical strategies: Examples include gaze stabilization with saccadic training exercises that can be performed with a laser on the head during walking or stair climbing in a more ecological setting. This approach ensures that rehabilitation becomes an integral part of the patient’s daily routine, leading to faster recovery and better long-term outcomes.
- Activities of daily living: It is important to return to work activities, even if they cause some discomfort. In the early stages of recovery, it may be helpful to engage in daily activities that previously caused gaze and postural instability, such as going to the supermarket, shopping, or returning to the workplace.
- Customized exercise programs: Tailoring exercise programs to each patient’s lifestyle is key to success. For example, patients who enjoy walking may benefit from incorporating balance challenges such as walking on uneven surfaces or turning their heads while walking. Younger, more active patients may integrate balanced exercises into sports or more dynamic activities.
- Reducing anxiety (fear of falls) and increasing motivation are two psycho-affective/emotional factors that must be considered in VPT. This is particularly relevant for patients with PPPD or residual dizziness.

## 5. Conclusions

The new approaches to VPT offer several advantages over traditional methods. Sensory substitution, behavioral strategies, and dynamic habituation techniques enhance compensation for vestibular deficits, providing patients with better gaze and postural stability. Earlier is better for optimizing functional recovery. Saccade training may address dynamic compensation, helping patients regain visual stability and adapt more efficiently to their environments. Dynamic postural stability training and cognitive–motor dual tasks further support recovery by improving postural control and cognitive flexibility, ensuring that patients are better equipped to manage complex real-world situations.

## Data Availability

No new data were created as part of this study.
